# Posttranslational Protein Modification in the Salivary Glands of Sjögren's Syndrome Patients

**DOI:** 10.1155/2013/548064

**Published:** 2013-03-05

**Authors:** Rafael Herrera-Esparza, Mayra Rodríguez-Rodríguez, María Elena Pérez-Pérez, Martha Adriana Badillo-Soto, Felipe Torres-del-Muro, Juan José Bollain-y-Goytia, Deyanira Pacheco-Tovar, Esperanza Avalos-Díaz

**Affiliations:** Department of Immunology, School of Biological Sciences, Universidad Autónoma de Zacatecas, Chepinque 306, Colonia Lomas de la Soledad, 98040 Zacatecas, ZAC, Mexico

## Abstract

The present study investigated posttranslational reactions in the salivary glands of patients with Sjögren's syndrome. We analysed the biopsies of primary Sjögren's patients using immunohistochemistry and a tag-purified anticyclic citrullinated protein (CCP) antibody to detect citrullinated peptides, and the presence of peptidylarginine deiminase 2 (PAD2) was assessed simultaneously. The present work demonstrated the weak presence of the PAD2 enzyme in some normal salivary glands, although PAD2 expression was increased considerably in Sjögren's patients. The presence of citrullinated proteins was also detected in the salivary tissues of Sjögren's patients, which strongly supports the *in situ* posttranslational modification of proteins in this setting. Furthermore, the mutual expression of CCP and PAD2 suggests that this posttranslational modification is enzyme dependent. In conclusion, patients with Sjögren's syndrome expressed the catalytic machinery to produce posttranslational reactions that may result in autoantigen triggering.

## 1. Introduction

Sjögren's syndrome is an autoimmune epithelitis that primarily affects the salivary and lachrymal glands and results clinically in Sicca syndrome, which is characterised by xerostomia, xerophthalmia, and keratoconjunctivitis [1].

Citrulline is an *α*-amino acid that can be isolated from watermelon [2]. In addition, it is an intermediate compound of the urea cycle and is synthesised in the liver by the conversion of ornithine to arginine during urea formation [3]. Citrulline exists in two forms: free citrulline, which is a product of the NOS enzyme family, and citrulline that results from the posttranslational modification of certain proteins at arginine residues, which is catalysed by peptidylarginine deiminases (PADs). This family of enzymes deiminates proteins at arginine residues to yield citrulline residues, and in this manner, protein citrullination is NOS independent [4].

We previously demonstrated inflammatory citrullination in the salivary glands of Sjögren's syndrome patients that was partially dependent on iNOS. This enzyme is overexpressed in inflammatory cells and infiltrates of the acini and salivary epithelia, and the *in situ* NO overdrive may contribute to glandular damage [5]. TNF and other proinflammatory cytokines are frequently observed in the glandular tissues of these patients, and these factors may trigger the production of iNOS [6]. Although protein citrullination is an important event in rheumatoid arthritis pathogenesis [7, 8], this process may also occur in Sjögren's syndrome. Approximately 7.5% of primary Sjögren's syndrome patients are positive for anticyclic citrullinated protein (anti-CCP) antibodies [9], and the presence of this marker in Sjögren's syndrome may be associated with nonerosive arthritis [10]. Protein citrullination has been shown to be an important pathophysiological mechanism in rheumatoid arthritis, and this process has also been reported in other autoimmune diseases such as antibody-associated neurodegenerative diseases [11]. Therefore, we investigated protein citrullination at the glandular level in Sjögren's syndrome.

## 2. Material and Methods

### 2.1. Tissues

Minor salivary gland biopsies from 24 patients with primary Sjögren's syndrome were studied. The patients fulfilled the revised version of the European criteria proposed by the American-European Consensus [12]. This study included 17 women and seven men with a mean age of 47 years (range, 37–57 years). All of the patients and controls were informed of the biopsy, and written authorisation was obtained. An equal number of biopsies were obtained during oral surgery for other purposes from individuals without Sjögren's syndrome, who were included as controls. The ethics committee of our institution authorised and monitored the protocol, which conformed to the requirements of the World Medical Association's Declaration of Helsinki. The specimens were fixed in formaldehyde, embedded in paraffin, sectioned at a 4 *μ*m thickness, and stained with haematoxylin and eosin. Two pathologists microscopically evaluated the biopsies, which were classified according to the revised multilevel assessment of a cumulative focus score [13]. Unstained tissue sections were used for immunohistochemistry.

### 2.2. Anti-CCP Antibodies

Enzyme immunoassays detected antibodies against citrullinated synthetic peptides from vimentin and filaggrin in sera from patients and controls. Serum from patients with rheumatoid arthritis was used as a positive control. The assays were performed according to the manufacturer's instructions (Euroimmun AG, Luebeck Germany). First, the serum samples were diluted 1 : 100 in dilution buffer. Second, the sera dilutions were incubated for 60 minutes at room temperature in wells of polystyrene plates coated with synthetic cyclic citrullinated peptides (CCPs), which contained arginine-modified residues. The citrullinated synthetic peptides included filaggrin, EBV nuclear antigen, and IgG sequences, and the CCPs included recombinant rat filaggrin or mutated human vimentin [14]. Third, the plates were washed with washing buffer. Fourth, the bound antibodies were tagged with the secondary antibody (conjugated POD-rabbit polyclonal anti-human IgG) during a 30-minute incubation. Fifth, the colour reaction was developed during a 30-minute incubation with the colour developer pNPP (*p*-nitrophenyl phosphate, disodium salt). Sixth, the reaction was stopped, and the microplates were evaluated using an ELISA reader at 450 nm. Lastly, five calibrators were used to construct the curve analysis, and the results are expressed in units. All assays were performed in triplicate.

### 2.3. Purification of Affinity-Bound CCP Peptides

A rheumatoid arthritis patient who was positive for anti-CCP antibodies (filaggrin or mutated human vimentin) was submitted to ELISA, as described previously [15]. Specific anti-CCP bound antibodies were eluted from the polystyrene CCP-coated plates after a 2-hour incubation using 0.2 M glycine-HCl, pH 2.8, and the eluted antibodies were neutralised with 1 M Tris, pH 9.5. The recovered antibodies were concentrated in a Centricon centrifugal device with a 30 kDa molecular weight cut-off (Millipore).

### 2.4. Antibodies Labelling with Peroxidase

Protein concentrations of affinity-purified antibodies were measured using the Bradford method [16], and the high-affinity-purified anti-CCP antibodies were labelled with horseradish peroxidase (HRP) (Sigma, St. Louis, MO) using the method described by Avrameas [17] with modifications. Briefly, the molar ratio of high-affinity-purified antibodies to peroxidase was 1 : 10. The enzyme was preactivated with glutaraldehyde and incubated at 37°C for 30 minutes. The coupling reaction was induced during an 18-hour incubation at room temperature in a sodium carbonate buffer, pH 9.5. The Anti-CCP/HRP was extensively dialysed against distilled H_2_O using a membrane with a pore size of 40 kDa. The anti-CCP/HRP conjugate was fractionated in a minicolumn packed with Sephadex G-200, which had been equilibrated with PBS, and an elution volume of 120 *μ*L per/fraction was collected in 25 tubes. The protein concentration was recorded in the different fractions, and three pools were created according to the chromatogram. Direct ELISA assays determined the reactivity to CCP of each pool, and each pool was concentrated to obtain 33% of the final volume using a Centricon device. The affinity anti-CCP-purified antibodies were used to trace CCP antigens in the salivary glands using immunohistochemistry.

### 2.5. Immunohistochemistry

Immunohistochemistry was performed to detect posttranslationally modified proteins in 4 *μ*m-thick sections of fixed minor salivary glands on microscope slides. The specimens were deparaffinised, permeabilised with 0.01% Triton X-100/phosphate-buffered saline (PBS), and washed three times with PBS. Endogenous peroxidase was quenched for 10 minutes using 3% H_2_O_2_ in methanol. The tissues were incubated with purified human IgG precipitated from normal human sera with ammonium sulphate to neutralise the presence of possible rheumatoid factor activity in salivary glands, and the precipitates were dialysed against distilled water using Slide-A-Lyzer Dialysis Cassettes (10 K MWCO Thermo Fisher Scientific Inc., Rockford, IL). IgG was purified using HiTrap protein G HP columns. The tissues were washed and incubated for 12 hours with an affinity-purified anti-CCP antibody (diluted 1 : 50) in 10% BFS-PBS. Bound anti-CCP antibodies were developed using 3,3′-diaminobenzidine-0.06% H_2_O_2_ (Sigma, St. Louis, MO) after washing, and the reaction was stopped with 0.5 M sulphuric acid. The slides were examined under a light microscope. All assays were performed in triplicate and evaluated in a blinded manner. In addition, an anti-citrulline antibody (cat. 231246, Calbiochem, Darmstadt, Germany; 1 : 100 dilution in 10% foetal bovine serum (FBS)-PBS) and an anti-PAD 2 antibody (PA5-19474, Pierce; diluted 1 : 30) were tested on salivary glands in immunohistochemistry assays. These antibodies were incubated overnight, washed with PBS, and incubated with the secondary antibody (goat anti-rabbit IgG labelled with peroxidase; Abcam ab759). The colour reaction was induced as previously described and evaluated in a blinded manner [5]. 

### 2.6. Statistical Analyses

Data were processed using a Fisher's exact test using the GraphPad Software, QuickCalcs; the two-tailed *P* value <0.005 was considered statistically significant.

## 3. Results

### 3.1. Lymphocyte Infiltrates

The absence of any inflammatory infiltrate was classified as a score of 0, which was characteristic of all control biopsies. Five biopsies from Sjögren's syndrome patients were scored as class I, and these biopsies exhibited discrete sialadenitis. Six biopsies were scored as class II and exhibited a moderate infiltration of mononuclear cells grouped in a single focus. Five biopsies were scored as class III and exhibited severe sialadenitis and one infiltrating focus. Eight biopsies were scored as class IV and displayed chronic sialadenitis with more than one foci (Table 1).

### 3.2. Purification and Labelling of High-Affinity Anti-CCP Antibodies

**Figure 1:**
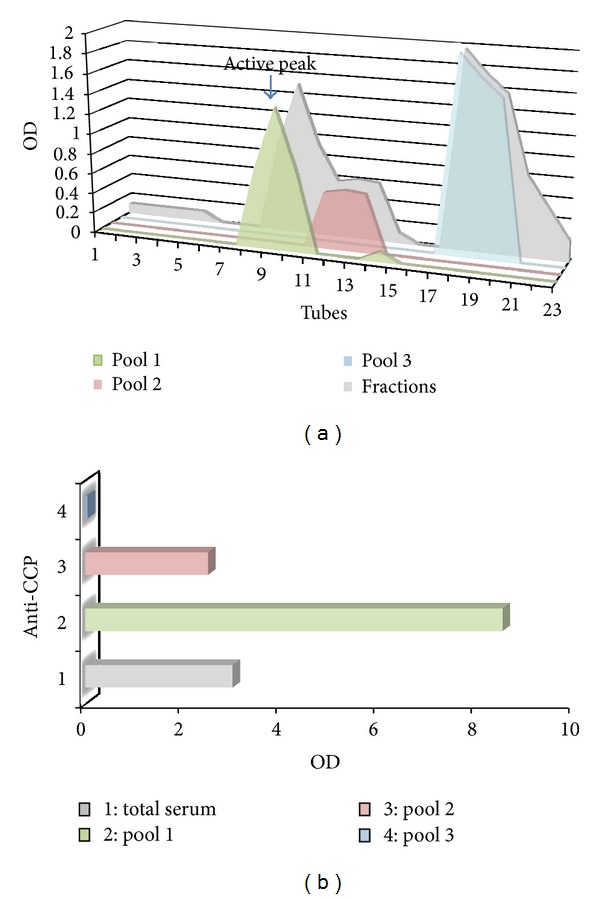
(a) Chromatogram shows the purification of anti-CCP conjugated antibody that was further fractionated using gel filtration chromatography, which produced three fractions. The first pool (tubes 9–11) contained the HRP-anti-CCP conjugates (green) second pool (tubes 12–14) (pink). Pool 3 (tubes 18–20) contained the nonconjugated peroxidase fraction (blue). (b) ELISA graph of different fractions obtained by gel filtration. Pool 1 was very reactive for CCP, with an optical density that was threefold higher than the original serum titre. Pool 2 activity was weaker, and pool 3 was negative. (OD: optical density).

We purified anti-CCP antibodies, which were bound to commercial polystyrene plates covered with citrullinated synthetic peptides, from the total protein in each microwell. We recovered 30 *μ*g/well of specific and high-affinity anti-CCP antibodies after glycine elution. We used the traditional coupling method of horseradish peroxidase bound to anti-CCP antibodies and glutaraldehyde at a molar ratio of enzyme: antibody of 10 : 1, which worked well. The conjugated antibody was further fractionated using gel filtration chromatography, which produced three fractions. The first fraction pool contained the HRP-anti-CCP conjugates (fractions 9–11 or pool 1), and this fraction was very reactive to CCP by ELISA, with an optical density that was threefold higher than the original serum titre. The anti-CCP activity was weaker in the second pool (fractions 12–14 or pool 2). Pool 3 (fractions 18–20) contained the nonconjugated peroxidase fraction, which was negative for anti-CCP by ELISA (Figure 1).

### 3.3. Anti-CCP Antibodies

Two of the samples from primary Sjögren's syndrome patients were positive for anti-CCP antibodies in the serum, whereas healthy controls were negative for anti-CCP antibodies. 

### 3.4. Normal Salivary Glands Express PAD2

The peptidylarginine deiminases belong to a family of enzymes that posttranslationally modify proteins at positively charged arginine residues to neutral citrulline residues via a citrullination process. These enzymes play a role in various physiological processes, and they are widely distributed in tissues. Therefore, we investigated the distribution of these enzymes in normal salivary glands and observed the discrete presence of PAD2 in the ductal epithelia of selected minor salivary gland of some biopsies. However, expression of this enzyme was absent in the acini.

### 3.5. Protein Posttranslational Modification Occurs in the Salivary Glands of Sjögren's Syndrome Patients

**Figure 2:**
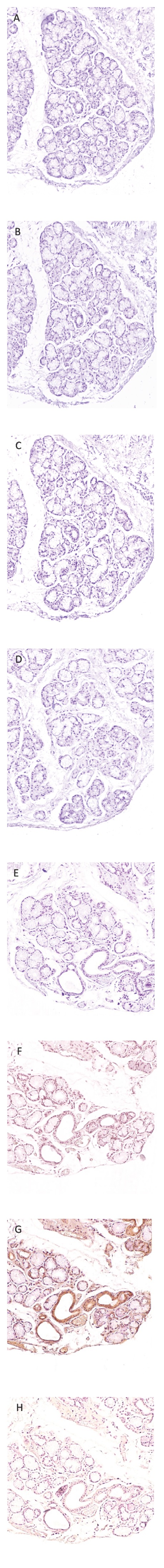
Immunohistochemistry of minor salivary glands. The superior panel corresponds to normal controls, and the inferior panel belongs to one representative biopsy of a Sjögren patient. (A) and (E). Incubated with PBS. (B) and (F). Treated with anti-CCP. (C) and (G). Anti-PAD2. (D) and (H). Anticitrulline. Observe the immunoreagents positivity at ducts and acini in (F), (G), and (H).

**Figure 3:**
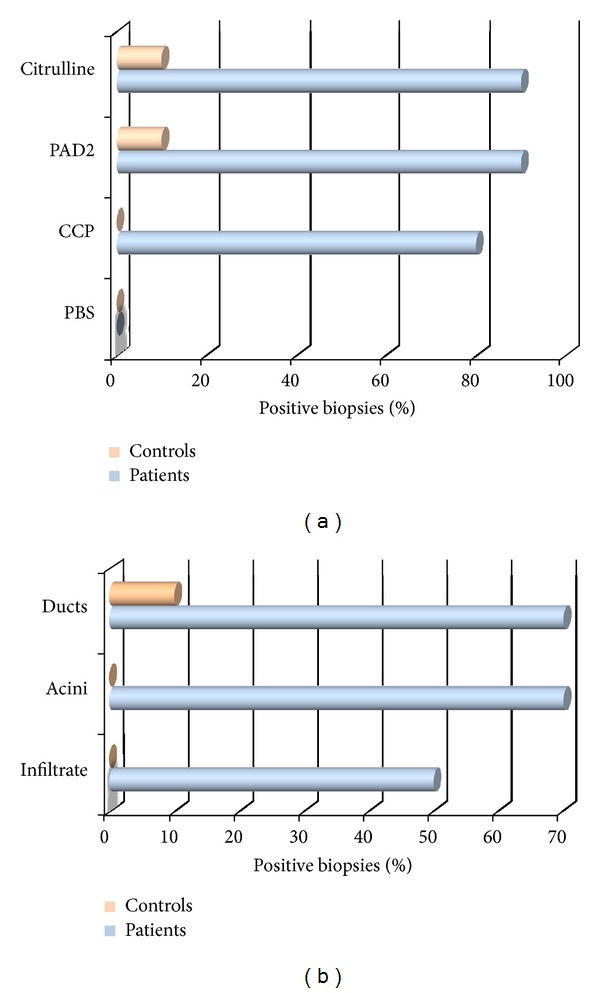
(a) Comparative immunohistochemistry assay of CCP, PAD2, and citrulline in controls and Sjögren patients; note that 10% of normal biopsies express the PAD2 enzyme, while Sjögren patients exclusively express CCP mutually distributed with PAD2. (b) Distribution of CCP/PAD2 in compartments of salivary glands of Sjögren patients.

We next examined the presence of posttranslationally citrullinated proteins in the salivary glands of primary Sjögren's syndrome patients using our high-affinity-purified anti-CCP antibodies. We detected the presence of posttranslationally modified proteins in the salivary ducts, acini, and inflammatory infiltrate foci of 70% of the Sjögren's syndrome patient biopsies, although evidence for this citrullination process was absent in normal control samples, *P* value <0.0001 (Figures 2 and 3 and Table 1).

### 3.6. Protein Citrullination Is PAD2 Dependent

**Figure 4:**
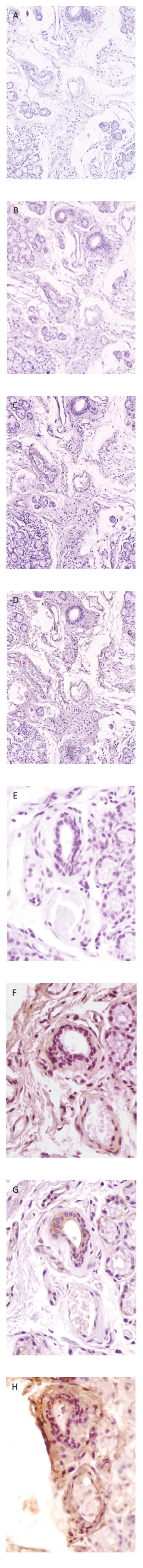
Immunohistochemistry of minor salivary glands from Sjögren patients; the superior panel shows an overview of immunoreagent distribution at 10x magnification; the inferior panel showed a close up at 40x magnification. (A) and (E). Incubated with PBS. (B) and (F). Treated with anti-CCP. (C) and (G). Anti-PAD2. (D) and (H). Anticitrulline. Note the correlation between CCP, PAD2, and citrulline expression in a duct.

The distribution of PAD2 was then compared to the distribution of citrullinated peptides (according to anti-CCP labelling) to determine the association between this enzyme and *in situ* citrullination. We observed total agreement between the presence of both factors, which suggested that this expression was consistent with the extensive areas of citrullination in the ducts, acini, and inflammatory infiltration foci. These results strongly support the *in situ* posttranslational modification of proteins (Figure 4).

## 4. Discussion

The antigenic triggering by posttranslational modifications of normal proteins is an important component of autoimmune diseases. This process transforms certain protein epitopes that may trigger an autoimmune response in genetically susceptible individuals. The present study investigated whether the salivary glands in Sjögren's syndrome patients are potential sources of posttranslational protein modification. Biopsies from primary Sjögren's syndrome patients were used to detect posttranslationally modified proteins using a tag-induced anti-CCP antibody and immunohistochemistry. The potential role of the PAD2 enzyme as the catalyst for these modifications was also assessed. The primary results of the present study can be summarised as follows. First, the PAD2 enzyme was detected in some normal salivary glands, but its expression was increased considerably in Sjögren's syndrome patients. Second, the presence of citrullinated proteins in the salivary tissues of Sjögren's patients strongly supports the posttranslational modification of proteins *in situ*. Third, the mutual expression of CCP and PAD2 suggests that posttranslational modifications are enzyme dependent. Finally, the inflammatory infiltrates were associated with PAD2 overexpression, which was directly related to the presence of protein modifications.

The PAD2 enzyme isoform is extensively distributed in a variety of mammalian tissues, including the nervous system, skeletal muscle, pancreas, sweet glands, salivary glands, mammary fat pads, bone marrow, and immune cells such as monocytes and macrophages [18–20]. The salivary glands broadly express proteins that may be modified by PAD, including vimentin, enolase, and others [21–23]. Furthermore, the salivary glands constitutively express PAD2, and this enzyme may convert arginine residues into citrulline, which is the cornerstone of the citrullination process. Additionally, the epithelial and glandular components of salivary glands express vimentin, enolase, and other proteins that are potential substrates for PAD2, and some of these proteins have been recognised as autoantigens in autoimmune diseases, including rheumatoid arthritis. Therefore, we assumed that all of the necessary elements for posttranslational modification existed, and these molecules were observed to be present in the salivary glands of patients with Sjögren's syndrome. Following this posttranslational process, modified proteins have been shown to trigger autoimmune responses. The first evidence for antibody generation in response to modified cellular components was reported as the presence of autoantibodies against filaggrin, which is detected in normal epithelial cells of the oral mucosa. Therefore, this autoantibody was termed as an “antiperinuclear factor” and was shown to be present in rheumatoid arthritis patients. Similarly, anti-CCP antibodies serve as an early marker of rheumatoid arthritis, and the importance of anti-CCP antibodies in disease pathogenesis has been increasingly demonstrated [24–28]. Furthermore, posttranslational protein modifications may trigger other autoimmune diseases, such as optic neuritis, coeliac disease, and multiple sclerosis [29–31].

The biggest challenge in the current study was the poor availability of anti-CCP reagents from a commercial source. Therefore, we obtained autoantibodies from rheumatoid arthritis patients to perform our studies. We were inspired by the study of Vossenaar et al. [32], as these authors obtained affinity-purified antibodies from the serum of rheumatoid arthritis patients using an immunoadsorption technique with recombinant peptides that were produced by phage display technology. We used a very simple technology that exploited the ability of immobilised synthetic peptides on polystyrene commercial plates to bind high-affinity anti-CCP antibodies, which were eluted from plates with a glycine buffer that lowers the pH. This method recovered a useful amount of high-affinity anti-CCP antibodies, which demonstrated a threefold increased reactivity to citrullinated synthetic peptides in commercial ELISA plates. These high-affinity anti-CCP antibodies were conjugated to peroxidase, which served as an excellent tag for CCP, as demonstrated in the immunohistochemistry assays; efficiency of our antibodies is comparable to that previously reported by other authors [32]. Furthermore, potential false positives for the presence of rheumatoid factor were prevented by the preincubation with normal IgG, which neutralised the rheumatoid factor activity.

Our results are important for understanding Sjögren's syndrome pathophysiology because these modifications constitute an additional mechanism for the triggering of autoimmunity. These modifications may also be applicable to other autoimmune diseases because posttranslational modification may represent a common mechanism for the genesis of autoantigens. For example, the study by Routsias et al. demonstrated that the posttranslational modification of the auto-antigen La increased its antigenicity [33, 34]. These results support a new perspective for exploring the origins of autoimmunity based on antigenic triggering in genetically susceptible individuals.

However, many questions remain unresolved. For example, do the constitutively expressed PAD enzymes in normal glandular epithelia (PAD2) play a role in the initial triggering of autoimmunity in Sjögren's syndrome? Additionally, do previous triggers induce inflammatory foci formation and PAD2 overexpression in mononuclear cells and macrophages leading to the posttranslational modification of salivary gland proteins? Both of these possibilities may coexist and create a feedback loop, although the theoretical aspects of this hypothesis require clarification using other experimental approaches.

## 5. Conclusions

In conclusion, Sjögren's syndrome patients possess the catalytic machinery to posttranslationally modify proteins, which may result in auto-antigen triggering.

## Figures and Tables

**Table 1:** Clinical, serologic, and immunohistochemistry data.

		Sjögren (*n* = 24)			Controls (*n* = 24)		
Clinical data							
Sicca		24 (8 = IV, 5 = III, 6 = II, 5 = I)			0		*P < 0.0001
Glandular		24 (8 = IV, 5 = III, 6 = II, 5 = I)			0		*P < 0.0001
Extraglandular		10 (8 = IV, 2 = III)			0		*P < 0.0006

Serology							
Anti-CCP		2 (2 = IV)			0		*P < 0.4890
+RF		10 (8 = IV, 2 = III)			0		*P < 0.0006
+ANA		16 (8 = IV, 5 = III, 3 = II)			0		*P < 0.0001
+Anti-La		12 (7 = IV, 4 = III, 1 = II)			0		*P < 0.0025
+Anti-Ro		14 (8 = IV, 4 = III, 1 = II, 1 = I)			0		*P < 0.0001

Biopsies							
	Acini	Ducts	Infiltrates	Acini	Ducts	Infiltrates	

PAD 2	17 ^*£*^(8 = IV, 6 = III, 2 = II, 1 = I)	17 ^*£*^(8 = IV, 6 = III, 2 = II, 1 = I)	12 ^*£*^(8 = IV, 4 = III)	0	2	0	*P < 0.0001
CCP	17 ^*£*^(8 = IV, 5 = III, 3 = II, 1 = I)	17 ^*£*^(8 = IV, 5 = III, 3 = II, 1 = I)	17 ^*£*^(8 = IV, 5 = III, 3 = II, 1 = I)	0	0	0	*P < 0.0001
Citrulline	19 ^*£*^(8 = IV, 5 = III, 5 = II, 1 = I)	19 ^*£*^(8 = IV, 5 = III, 5 = II, 1 = I)	17 ^*£*^(8 = IV, 5 = III, 3 = II, 1 = I)	0	2	0	*P < 0.0001

*Sjögren versus controls.

^*£*^Sjögren class IV versus class I = *P* < 0.003.

Histology class inside the parentheses.
